# Note on the Use of the New Local Anæsthetic

**Published:** 1851-06

**Authors:** T. D. Mütter

**Affiliations:** Professor of Surgery in Jefferson Medical College, Philadelphia


*Note on the Use of the new local Anæsthetic.* By T. D. Mütter, M. D., Professor of Surgery in Jefferson Medical College, Philadelphia.

I am indebted to my friend Mr. Barnet Phillips, of Philadelphia, for a specimen of the new local anæsthetic, known to us by the several terms, "Eau Hollandaise," "Chlorure of the oil of the Dutch Chemists," and "Ether chlorohydrique chloré."

Mr. Phillips is now the *chief director* in the great establishment of Pelletier of Paris, and his high reputation as a manufacturing chemist, is a sufficient guarantee that the article sent is pure, and of the first quality.

Prepared, by the reports contained in some of the French journals, to find in this agent the much looked for *efficient* yet *harmless* local anæsthetic, I must confess that my disappointment was great when I discovered, by some experiments, that, in my hands at least, it failed to accomplish the object for which it was applied.

The local impression, indeed, was not greater than that produced by *chloroform*, and by no means so decided as that occasioned by *ice*.

